# Structural determinants of tailored behavioral health services for sexual and gender minorities in the United States, 2010 to 2020: a panel analysis

**DOI:** 10.1186/s12889-022-14315-1

**Published:** 2022-10-12

**Authors:** Cory J. Cascalheira, Emily C. Helminen, Thomas J. Shaw, Jillian R. Scheer

**Affiliations:** 1 Department of Counseling & Educational Psychology, New Mexico State University, 1780 E University Ave, Las Cruces, NM 88003 USA; 2 Department of Psychology, Syracuse University, 414 Huntington Hall, Syracuse, NY 13244 USA

**Keywords:** Stigma, Structural stigma, LGBTQ, Mental health, Substance use, Healthcare access, Panel data, Longitudinal, N-SSATS, N-MHSS

## Abstract

**Background:**

Research indicates that tailored programming for sexual and gender minority (SGM; e.g., lesbian, gay, bisexual, transgender, queer) people, compared to non-tailored programming, is effective for reducing the disproportionate health burden SGM people experience relative to the general population. However, the availability of SGM-tailored programming is often over-reported and inconsistent across behavioral health (i.e., substance use and mental health) facilities in the United States (U.S.).

**Methods:**

Using panel analysis, the National Survey of Substance Abuse Treatment Services (N-SSATS), and the National Mental Health Services Survey (N-MHSS), this study examines structural stigma and government funding as two structural determinants affecting the availability of SGM-tailored programming in the U.S.

**Results:**

Results indicated that from 2010 to 2020, reductions in structural stigma (i.e., increases in state-level supportive SGM policies) were positively associated with increases in the proportion of substance use treatment facilities offering SGM-tailored programming. This effect was significant after controlling for over-reporting of SGM-tailored programming and time- and state-specific heterogeneity. On average, the effect of reduced structural stigma resulted in approximately two new SGM-tailored programs in the short term and about 31 new SGM-tailored programs in the long term across U.S. substance use treatment facilities. Structural stigma did not predict the availability of SGM-tailored programming in mental health treatment facilities. Government funding was not significant in either data set. However, without correcting for over-reporting, government funding became a significant predictor of the availability of SGM-tailored programming at substance use treatment facilities.

**Conclusions:**

Because SGM-tailored programming facilitates access to healthcare and the current study found longitudinal associations between structural stigma and the availability of SGM-tailored programming in substance use treatment facilities, our findings support claims that reducing structural stigma increases access to behavioral health treatment specifically and healthcare generally among SGM people. This study’s findings also indicate the importance of correcting for over-reporting of SGM-tailored programming, raising concerns about how respondents perceive the N-SSATS and N-MHSS questions about SGM-tailored programming. Implications for future research using the N-SSATS and N-MHSS data and for public health policy are discussed.

**Supplementary Information:**

The online version contains supplementary material available at 10.1186/s12889-022-14315-1.

Compared to heterosexual and cisgender people, sexual and gender minority (SGM; e.g., lesbian, gay, bisexual, transgender, queer) people disproportionately experience poor behavioral health outcomes (e.g., substance use, mental health conditions). In terms of substance use, for example, a study using the 2015 National Survey on Drug Use and Health indicated that 39.1% of sexual minority adults (compared to 17.1% of heterosexual adults) used an illicit substance in the past year [1]. Compared to cisgender youth, transgender youth are 2.5 to 4 times more likely to report substance use [2]. Disparities in mental health among adult SGM populations are also evident [3–7]. To address these deleterious health outcomes, SGM-tailored programming for behavioral health treatment has been developed. SGM-tailored programming consists of substance use and mental health interventions targeting SGM people’s unique needs (e.g., minority stressors, such as family or peer rejection, victimization, and rejection hypervigilance) that are related to adverse behavioral health outcomes [8]. Despite its clinical effectiveness [9], SGM-tailored programming is unavailable to many SGM people because, as of 2018, only 19.9% of substance use treatment facilities and 17.7% of mental health treatment facilities reported offering SGM-tailored programming [10, 11]. While studies have examined determinants of SGM-tailored programming availability at the individual facility level, fewer have examined structural determinants (e.g., structural stigma, government funding) that may affect the availability of SGM-tailored programming.

## SGM-tailored programming at behavioral health facilities

SGM-tailored programming has demonstrated improvements in alcohol use, smoking, and other drug use among numerous SGM subgroups [9, 12–14]. For instance, one study found that a smoking cessation intervention among SGM young adults that included SGM-tailored programming in addition to cognitive-behavior therapy strategies demonstrated greater likelihood of smoking reduction and cessation when compared to an intervention implementing cognitive-behavioral therapy strategies alone [14]. The importance of SGM-tailored programming also extends to mental health treatment. Studies implementing SGM-tailored mental health treatments have demonstrated improvements in depression, anxiety, and self-efficacy among various SGM groups [9, 12, 15].

Two national, census-based surveys in the United States (U.S.) track the availability of SGM-tailored programming: the National Survey of Substance Abuse Treatment Services (N-SSATS) [16] and the National Mental Health Services Survey (N-MHSS) [17]. Several studies have used these data to examine facility-level descriptions of SGM-tailored programming across the U.S. [10, 11, 18]. For example, evidence indicates that SGM-tailored programming is associated with specific facility characteristics [11, 18], such as a facility’s ownership, geographic location, whether it receives government funding, and the type of services offered (e.g., medication management vs. outpatient only).

Understanding facility-level associations can reveal targetable, structural determinants for public health policymakers. For example, since substance use treatment facilities that receive federal funding are more likely to offer SGM-tailored programming [11], public health policymakers could mandate federally funded facilities to offer such programming [19]. However, because public health policy and governing structures vary by state [20], and government funding for substance use and mental health treatment includes state and local funds [16, 17], there is a need to understand state-level structural determinants that may influence the availability of SGM-tailored programming.

## Structural stigma and government funding as structural determinants

Two structural determinants that may affect the availability of SGM-tailored programming are structural stigma and government funding. *Structural stigma* is conceptualized as the social conditions, cultural norms, and institutional policies that detract from the wellbeing of marginalized communities, including SGM people [21]. Research has primarily examined structural stigma as directly impacting SGM people’s health [22–26], but structural stigma also limits access to high-quality social resources [27], such as healthcare. SGM people living in areas with high structural stigma (i.e., U.S. Census regions with more anti-SGM policies) are less likely to access healthcare [28] and more likely to report lower-quality care [29]. Fortunately, structural stigma is inversely associated with SGM access to behavioral health services. For example, sexual minority youth of color living in states with lower structural stigma (e.g., fewer homophobic cultural norms) report more consistent access to behavioral health treatment compared to sexual minority youth of color living in states with higher structural stigma [30].

Government funding also is an important structural determinant influencing health outcomes and the availability of healthcare services. Evidence indicates that increases in government expenditures are associated with decreases in deleterious health outcomes [31, 32]. Funding allocations for healthcare services can be politicized [33–35] as government budgets are determined by political parties, and political parties differ in their support for SGM civil rights between parties and over time (i.e., differ in how they enact structural stigma) [36]. Hence, as political parties gain power, their annual budget allocations could reflect anti-SGM funding priorities. For example, when the Trump Administration proposed cutting $839 billion in Medicaid funding, SGM people and other historically disadvantaged groups were most likely to be affected [37]. In 2021, Florida Gov. Ron DeSantis cut funding for SGM mental health programs [38].

Yet, it is unclear whether government funding is merely an indicator of structural stigma or a unique (non-anti-SGM) structural determinant affecting the availability of SGM-tailored programming. For example, government spending on health is also related to other factors (e.g., poverty, county population size) beyond political influence [34]. That is, even though substance use facilities receiving government funding were less likely to offer SGM-tailored programming in 2016 [18], it is unclear whether this association might be attributable to political influence (i.e., structural stigma enacted through government funding) or some other aspect of government funding (i.e., funding allocations based on county population size). Further, extant literature currently lacks an understanding of *how* government funding affects the availability of SGM-tailored programming. Examining government funding separately from and concurrently with structural stigma is important. For instance, by including both variables as predictors, isolation of a potential non-political-influence effect of government funding on the availability of SGM-tailored programming is possible.

## The benefits of a panel analysis approach

Although extant studies have advanced knowledge of factors influencing the availability of SGM-tailored programming, most studies have used cross-sectional designs that measure stigma at one timepoint [8, 18]. These results may be insufficient because “current manifestations of” structural stigma “are deeply embedded in historical processes” ([21], p. 2). Conversely, longitudinal designs provide evidence for how structural stigma impacts SGM health over time, allowing researchers to make temporal, quasi-causal claims. For example, one study demonstrated that over a four-year period, sexual minority youth living in states with lower structural stigma (versus higher structural stigma) were significantly less likely to have smoked tobacco in the past year, even after controlling for individual (e.g., race) and state-level confounders (e.g., state smoking prevalence) [39]. As such, longitudinal studies examining the influence of structural stigma and government funding on the availability of SGM-tailored programming are warranted.

Two studies have used longitudinal approaches to examine the availability of SGM-tailored programming. The first study by Qeadan et al. [11] used the N-SSATS data to show that SGM-tailored programming increased from 2008 to 2018 and was associated with facility characteristics (e.g., more likely to be owned by the federal government). However, because there was no linking identifier for facilities across survey waves, it was not possible to examine whether these associations changed over time. By aggregating facilities to the state-level, the present study addresses this problem. We also extend Qeadan et al.’s [11] findings by using panel analysis—a rigorous longitudinal approach that can estimate the proportion of a state’s treatment facilities offering SGM-tailored programming across time [40].

Although the second longitudinal study examining the availability of SGM-tailored programming used panel data [10], the present study also builds upon this work in several ways. First, Chen and colleagues examined the N-MHSS data from 2014 to 2018, whereas we examine an additional year (i.e., 2019) and also investigate the N-SSATS data from 2010 to 2020. By examining SGM-tailored programming over longer periods, we potentially reduced the bias and inconsistency of estimates from the panel models [40]. Second, Chen et al. [10] found that the odds of offering SGM-tailored programming at mental health treatment facilities decreased by 10% annually, but determinants predicting these decreases were unclear. We extend this work by examining two structural determinants of SGM-tailored programming availability over time. Finally, it is unclear whether Chen et al. [10] leveraged the numerous methodological benefits of panel analyses relative to traditional time-series designs ([40], p. 6), such as (1) controlling for both state- and time-specific heterogeneity (i.e., controlling systematic differences between states and across years), (2) producing more reliable estimates by increasing variability and decreasing collinearity (i.e., increasing confidence in the results and reducing error), and (c) studying dynamics (i.e., how SGM-tailored programming in the previous year may be related to SGM-tailored programming in the present year). The present study implements each of these methodological advantages.

## The present study

Given that the reduction of adverse health outcomes is a public health priority [41] and SGM-tailored programming may be an effective method for achieving this aim [14], the current study used panel analysis to investigate whether structural stigma and government funding were associated with the availability of SGM-tailored programming across U.S. behavioral health facilities. We expected increases in state-level supportive SGM policies (i.e., decreases in structural stigma) to be associated with a greater proportion of behavioral health facilities offering SGM-tailored programming. Similarly, we hypothesized that increases in government funding would be associated with a greater proportion of behavioral health facilities offering SGM-tailored programming.

## Method

### Measures

All measures except for structural stigma come from the N-SSATS and N-MHSS data sets. The N-SSATS and N-MHSS data are national, census-based surveys of substance use and mental health treatment facilities administered by the Substance Abuse and Mental Health Services Administration (SAMSHA). With these surveys, SAMHSA attempts to collect information on the location, characteristics of, and services offered by all behavioral health facilities in the United States annually. Based on the annual reports of each survey, the average response rate (*M* = 85.72%) for the N-MHSS and the average response rate (*M* = 92%) for the N-SSATS exceeded the 80% threshold typically deemed to reduce the chances of selection bias [42]. For year-by-year response rates, see the Supplemental Materials (p. 3).

#### SGM-tailored programming at substance use treatment facilities

SGM-tailored programming for substance use was extracted from the 2010–2020 N-SSATS data sets. An average of 14,221 (*SD* = 916) substance use treatment facilities participated in the N-SSATS each year. First, the state variable was used as the spatial unit of analysis to which facility-level data were aggregated. Only 50 states were used to combine the N-SSATS variables with the Movement Advancement Project (MAP) data (i.e., Puerto Rico not included). Second, a variable was used to indicate whether a substance use treatment facility offered SGM-tailored programming (1 = facility offered SGM-tailored programming; 0 = did not offer). Since SGM-tailored programming was at the facility-level, it was aggregated into state-level counts to match the spatial unit of analysis in the MAP data. The counts were transformed into percentages (for an explanation, see Supplemental Materials, p. 2), where higher scores indicate more SGM-tailored programming at substance use treatment facilities.

#### SGM-tailored programming at mental health treatment facilities

SGM-tailored programming for mental health was taken from the 2014–2019 N-MHSS data sets. An average of 12,186 (*SD* = 611) mental health treatment facilities participated in the N-MHSS each year. Although the N-MHSS was published in 2010, the year 2014 was selected because it is the first year that SAMSHA began to publish annual reports (i.e., prior to 2014, the data were published every other year, and including 2010 and 2012 would affect model performance) [40]. First, state names were used to aggregate facility-level data. Second, SGM-tailored programming was used to indicate whether a mental health treatment facility offered SGM-tailored programming (1 = offered SGM-tailored programming; 0 = did not offer). SGM-tailored programming was aggregated into counts and transformed into percentages, where higher scores indicate more SGM-tailored programming at mental health treatment facilities.

#### Structural stigma

Structural stigma (i.e., state-level supportive SGM policies) was assessed with MAP [43], which is an index on the degree to which a state enacts supportive SGM policies and has been used in previous work [22, 44, 45]. Specifically, MAP tracks legislation relevant to SGM civil rights (e.g., anti-discrimination or adoption laws) and rates the degree to which states enact such laws. For instance, a state receives a higher anti-discrimination rating (i.e., a higher pro-SGM rating) if it forbids discrimination based on sexual orientation and gender identity, a lower rating if it forbids discrimination based on sexual orientation only, and the lowest rating if it enacts no protections for SGM people. Given that MAP altered their policy tracking strategy over time in response to emergent legislative initiatives, the total policy score for each year was transformed using the percent of maximum possible (“POMP”) method [46] (see the Supplemental Materials, pp. 2–3). Higher scores indicate higher ratings of state-level pro-SGM policies (i.e., lower structural stigma).

#### Government funding

Government funding was operationalized using data from the 2010–2020 N-SSATS and the 2014–2020 N-MHSS datasets. From the N-SSATS, a government funding variable was extracted to indicate whether a facility received any federal, state, county, or local funds (1 = facility received government funding; 0 = did not receive). A set of six N-MHSS variables (see Supplemental Materials, p. 2) were used to create the government funding variable. If the facility reported at least one source of funding from these state, county, or local sources, it was coded as receiving government funding (1 = facility received government funding; 0 = did not receive). Government funding was aggregated into state-level counts and transformed into percentages, where higher scores indicate more government funding.

### Data analytic plan

Importantly, as previously mentioned, we aggregated facility-level data to the state level so all variables were at the same level for panel analysis. However, because neither the N-SSATS nor the N-MHSS data sets have facility-level linking identifiers [11], aggregation was also necessary due to the structure of the data.

Data were analyzed in R 4.0.5 [47] and StataBE version 17. All code and data are available [48]. N-SSATS government funding data were missing for the year 2014, so values were replaced with expectation maximization using the R package *Amelia II* [49]. Expectation maximization also was used to replace New Hampshire’s missing data for SGM-tailored programming in the year 2012. No data were missing in the N-MHSS data sets. All variables were on a 0–100 percentage scale to ease interpretation.

Two dynamic panel, autoregressive-distributed lag (ADL [1, 1]) regression models with fixed effects—one for the N-SSATS data and one for the N-MHSS data—were estimated [40] to identify the relationship between the percentage of behavioral health facilities offering SGM-tailored programming within a state, the rating of state-level supportive SGM policies (i.e., structural stigma), and the percentage of behavioral health facilities within a state receiving government funding. Lagged dependent and independent variables were included in the model. A fixed-effects model was chosen to account for unobserved time-invariant characteristics and since no key variables were time-invariant. The final ADL(1, 1) models were created after examining assumptions, which are presented in the Supplemental Materials (p. 7–22). We controlled for unmodelled time-specific annual trending and for unobserved unit-specific effects. For full model specification, see the Supplemental Materials (pp. 24–25).

Because the panels were short (i.e., for the N-SSATS, the total number of states was 50 and the total number of time points was 10; for the N-MHSS the total number of states was 50 and the total number of time points was 6), the orthogonal reparameterization (OPM) estimator [50] was used to estimate the models. Pickup and Hopkins [51] conducted Monte Carlo simulations to show that, compared to the other estimators designed for short panels, the OPM estimator has the best properties for *N* $\le$ 100 (e.g., low bias on the long-run effects, greater efficiency across coefficients) and is robust to violations of distributional assumptions (for additional justifications, see Supplemental Materials, p. 25). OPM was implemented using the R package *OrthoPanels* [52]. Significance of the posterior parameter estimates was established with the 95% credible interval (CI), which is the Bayesian equivalent of the 95% confidence interval. *OrthoPanels* yields the short-run effect of the posterior parameters, which then is used to calculate the long-run effect. A short-run effect is the immediate impact of a predictor and the long-run effect is the cumulative influence of a predictor over multiple years [53].

#### Sensitivity analysis

Because there is significant uncertainty in the N-SSATS dependent variable, we performed a sensitivity analysis [54]. That is, Ji [55] and Cochran et al. [8] found that 70.8%–82.6% of substance use facilities who reported offering SGM-tailored programming in the N-SSATS actually did *not* offer these services. Thus, we sought to compare results from two models using the N-SSATS data: a model with a corrected versus a model with an uncorrected estimate of SGM-tailored programming at substance use treatment facilities.

We used Ji’s [55] data to correct the estimate of SGM-tailored programming at substance use treatment facilities because, compared to Cochran et al.’s [8] data, Ji’s data were most recent. Ji and a team of trained research assistants conducted structured telephone surveys with substance use facilities reportedly offering SGM-tailored programming. The team was trained on a structured telephone interview until consensus was reached on how to rate each facility during a phone call. The researchers called each facility between June 2020 and January 2021 and pretended to be someone seeking treatment services for a loved one who identified as SGM. Each facility was called at least three times before being marked as unreachable. Ji [55] was able to reach 1,811 of the 2,553 (70.9%) facilities listed in the 2018 N-SSATS dataset that claim to offer SGM-tailored treatment. The research team developed a codebook to categorize each facility’s SGM-tailored programming. The interrater reliability estimate of categorizing facilities based on the codebook was excellent (98.5%) [55]. Results indicated that only 315 facilities offered SGM-tailored programming; 1,496 did not. To be categorized as offering SGM-tailored programming, facilities either dedicated their entire program to SGM behavioral treatment (*n* = 12), had a sub-program specifically for SGM clients (*n* = 23), provided a specific tailored serviced (e.g., SGM support group, SGM housing; *n* = 135), individualized therapy for SGM clients (*n* = 73), or provided regular and mandatory provider training on SGM issues in therapy (*n* = 72). To be categorized as offering no SGM-tailored programming, the facility could not be classified into any of the above categories and either did not offer a specific SGM-tailored program (*n* = 1,273), stated their SGM-tailored programming was limited to non-discrimination policies (*n* = 80), indicated they did not accept SGM clients (*n* = 73), reported having SGM clients but no specific services (*n* = 64), or stated that they had SGM-tailored programming in the past or plan to offer SGM-tailored programming in the future but not currently (*n* = 6).

Given the robustness of Ji’s [55] method, a proportion was calculated (actual / reported SGM-tailored programming) to correct the estimate of SGM-tailored programming at substance use treatment facilities. We adjusted each state’s number of substance use facilities offering SGM-tailored programs for the years 2010 to 2020 by taking the product of this proportion (actual / reported number of SGM-tailored programming) times the reported number of SGM-tailored programming. The corrected number of facilities offering SGM-tailored programming was divided by the total number of substance use facilities to yield the new, corrected percentage of facilities offering SGM-tailored programming.

## Results

**Fig. 1:**
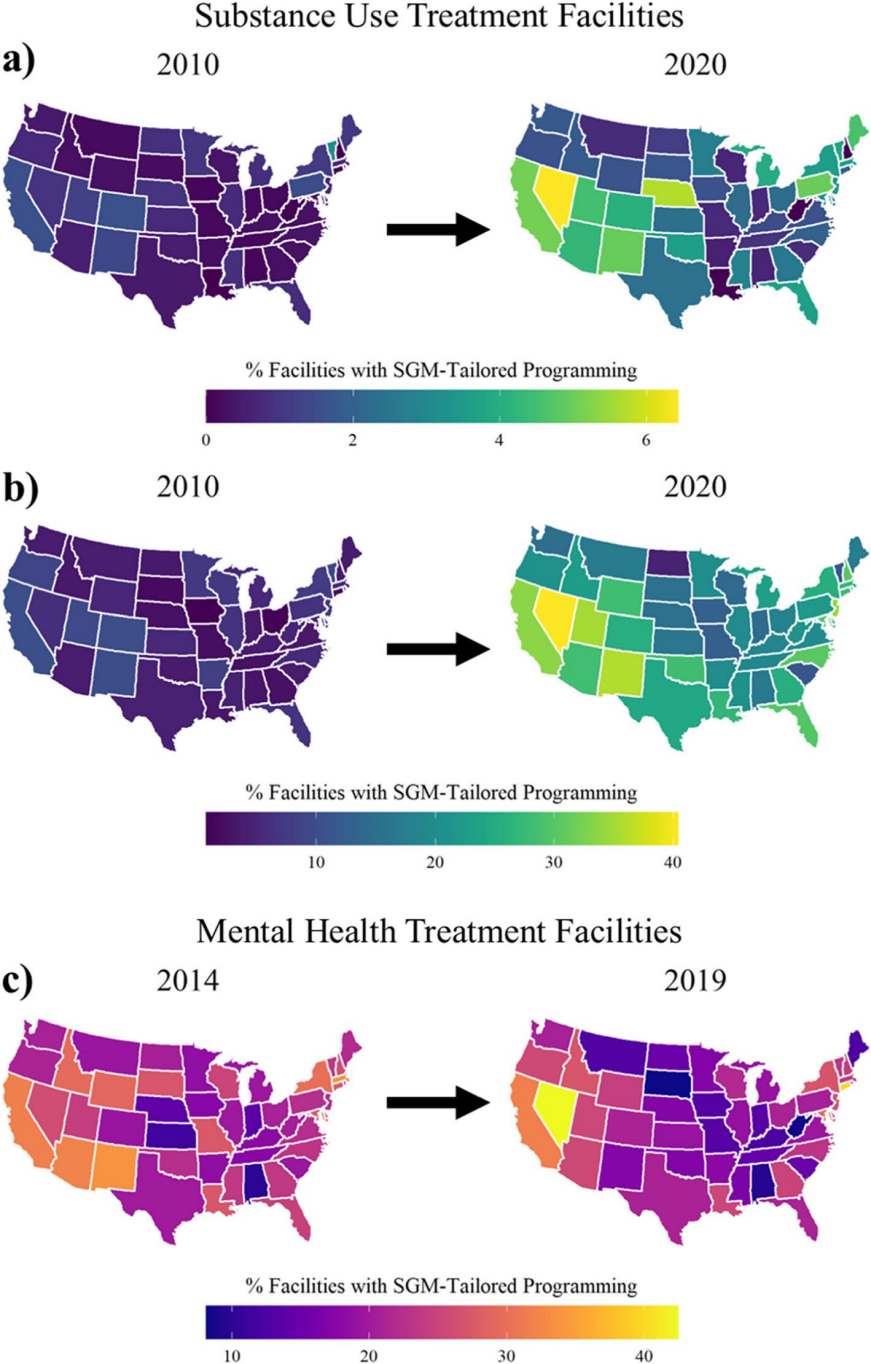
Percentages of Behavioral Health Facilities Offering SGM-Tailored Programming by State. Note. Visualizations depicting the **a** the Ji [55] corrected percentage of SGM-tailored programming at substance use treatment facilities, **b** the percentage of SGM-tailored programming reported by the N-SSATS, and **c** the percentage of SGM-tailored programming at mental health treatment facilities reported by the N-MHSS

Table 1 presents annual, descriptive characteristics of variables for substance use and mental health treatment facilities. In the uncorrected N-SSATS data, SGM-tailored programming at substance use treatment facilities increased from 5.01% to 22.14% from 2010 to 2020; using the Ji [55] correction, the increase was from 0.61% to 2.40%. From 2014 to 2019, SGM-tailored programming at mental health treatment facilities decreased from 22.89% to 20.58%. State-level differences in the percentage of behavioral health facilities offering SGM-tailored programming are depicted in Fig. 1. Additional descriptive statistics by year are presented in the Supplemental Materials (pp. 5–8).

**Table 1:** Annual Descriptive Characteristics of Variables

Substance Use Treatment Facilities (N-SSATS)				
Year	SGMP *M* (*SD*)	SGMP* *M* (*SD*)	SLP *M* (*SD*)	GVF *M* (*SD*)
2010	5.01 (2.48)	0.61 (0.58)	20.67 (12.86)	63.27 (12.45)
2011	4.22 (2.39)	0.52 (0.51)	29.35 (21.35)	62.87 (11.94)
2012	5.56 (2.75)	0.63 (0.51)	35.26 (28.69)	61.20 (12.19)
2013	10.54 (4.47)	1.19 (0.88)	42.62 (33.77)	60.63 (12.90)
2014	15.09 (5.34)	1.74 (1.24)	42.53 (35.67)	58.38 (13.14)
2015	15.79 (5.30)	1.79 (1.21)	27.58 (23.85)	57.99 (12.91)
2016	16.31 (5.99)	1.82 (1.27)	28.00 (25.74)	55.35 (14.03)
2017	17.12 (6.53)	1.96 (1.39)	32.43 (27.03)	56.60 (13.33)
2018	18.45 (6.55)	2.12 (1.60)	35.51 (28.34)	56.98 (13.31)
2019	20.57 (6.61)	2.32 (1.63)	39.35 (31.16)	55.92 (13.29)
2020	22.14 (7.47)	2.4 (1.65)	47.03 (31.92)	55.36 (13.32)
Mental Health Treatment Facilities (N-MHSS)				
Year	SGMPm *M* (*SD*)	SLP *M* (*SD*)	GVFm *M* (*SD*)	ΔGVFm *M* (*SD*)
2014	22.89 (5.38)	42.53 (35.67)	86.50 (6.92)	NA^a^
2015	17.09 (5.33)	27.58 (23.85)	85.69 (7.23)	$-$ 0.04 (9.9)
2016	12.40 (4.73)	28.00 (25.74)	85.66 (6.96)	0.03 (9.85)
2017	15.85 (6.34)	32.43 (27.03)	85.94 (6.54)	0.04 (8.86)
2018	17.84 (5.29)	35.51 (28.34)	85.57 (6.74)	$-$ 0.04 (9.48)
2019	20.58 (6.88)	39.35 (31.16)	75.86 (9.29)	$-$ 0.09 (12.27)

Mean and standard deviations of variables aggregated for each year across states. SGMP* indicates the Ji [55] correction to the percentage of substance use treatment facilities offering SGM-tailored programming. SLP indicates structural stigma. SGMP (substance use treatment facilities offering SGM-tailored programs), SGMP*, SGMPm (mental health treatment facilities offering SGM-tailored programs), GVF (substance use treatment facilities receiving government funding), and GVFm (mental health treatment facilities receiving government funding) are percentages. Delta (Δ) is the first difference of GVFm

^a^Indicates no first difference for the first year in the data set

See Table 2 for results from the OPM estimation of the ADL(1, 1) model for substance use treatment facilities (i.e., the N-SSATS data). In the model with the corrected estimate of SGM-tailored programming at substance use treatment facilities, the median posterior estimate for *ρ* was 0.935 (95% CI [0.821, 0.993]). The median posterior estimate for structural stigma was positive and significant, indicating that as a state enacts more pro-SGM policies (i.e., structural stigma reduces), the percentage of substance use treatment facilities with SGM-tailored programming within that state increases by 0.007% (95% CI [0.003, 0.012]). This means that, on average, roughly two new SGM-tailored programs at substance use treatment facilities are created in the short-term (i.e., within the same year) when reductions of structural stigma occur within a state. While the magnitude of the increase was small in the short-term, over time, the long-run effect of a state enacting pro-SGM policies increases the percentage of substance use treatment facilities with SGM-tailored programming within that state by 0.109% (95% CI [0.029, 0.991]). That is, on average and over time, roughly 31 new SGM-tailored programs at substance use treatment facilities are created when structural stigma is reduced.

**Table 2:** Orthogonal Reparameterization Results for the N-SSATS ADL(1, 1) Model

Variable	Short-Run Effect		Long-Run Effect	
	Med	95% CI	Med	95% CI
${\mathrm{SLP}}_{i,t}$	**0.007**	**[0.003, 0.012]**	**0.109**	**[0.029, 0.991]**
${\mathrm{SLP}}_{i,t-1}$	**0.004**	**[0.0004, 0.007]**	**0.058**	**[0.003, 0.681]**
${\mathrm{GVF}}_{i,t}$	$-$ 0.006	[$-$ 0.013, 0.001]	–0.088	[–0.949, 0.015]
${\mathrm{GVF}}_{i,t-1}$	$-$ 0.0002	[$-$ 0.006, 0.006]	–0.004	[–0.241, 0.309]

All values are percentages. Results were calculated using the Ji [54] corrected dependent variable. Med is the median of the distribution of the posterior parameter estimates. 95% CI is the 95% credible interval. Boldface indicates a significant estimate. SLP indicates structural stigma. GVF indicates substance use treatment facilities receiving government funding. Subscript *i* indicates the state, subscript *t* indicates the year. See the Supplemental Materials for more details on the subscripts

Results also indicated that reductions in structural stigma that occur via the enactment of pro-SGM state policies have a significant lagged effect. Pro-SGM policies from the year before are significantly, positively associated with an immediate 0.004% (95% [0.0004, 0.007]) increase in SGM-tailored programming. Over time, the long-run effect of last year’s pro-SGM policies is associated with a 0.058% increase (95% CI [0.003, 0.681]) in SGM-tailored programming. In other words, pro-SGM state policies enacted last year have a significantly positive immediate and cumulative impact on the availability of SGM-tailored programming at substance use treatment facilities, even after controlling for pro-SGM policies that were enacted in the current year. The median posterior estimates for government funding were not significant.

The sensitivity analysis revealed substantial differences between models with and without the corrected estimate of SGM-tailored programming at substance use treatment facilities. When the uncorrected dependent variable was used (i.e., the number of facilities with SGM-tailored programming reported in the N-SSATS$$)$$, *ρ* decreased to 0.771 (95% CI [0.633, 0.979]), the magnitudes of the median posterior estimates increased, and government funding became significant. The short-run effect of structural stigma exhibited an eight-fold increase to 0.062% (95% CI [0.030, 0.096]) and the long-run effect of structural stigma increased to 0.278% (95% CI [0.110, 3.25]). The median posterior estimate of the lagged effect of structural stigma increased to 0.030% (95% CI [0.003, 0.058]) in the short term and increased to 0.133% (95% CI [0.012, 2.004]) in the long term. That is, without using the corrected estimate of SGM-tailored programming at substance use treatment facilities, structural stigma exerted a substantially greater effect on the availability of SGM-tailored programming at these facilities. Regarding government funding, the short-run effect became $$-$$ 0.052% (95% CI [$$-$$ 0.103, $$-$$ 0.001]) and the long-run effect became $$-$$ 0.236% (95% CI [$$-$$ 2.048, $$-$$ 0.005]). These results indicate that as government funding for substance use treatment facilities increases within a state, the availability of SGM-tailored programming at substance use treatment facilities decreases in that state within the year and over time.

Table 3 presents the OPM results for the ADL(1, 1) model for mental health treatment facilities (i.e., the N-MHSS data). The median posterior estimate for *ρ* was 0.578 (95% CI [0.434, 0.753]). However, none of the variables were significant because the median posterior estimates all had 95% credible intervals that included zero. A longitudinal relationship between government funding and structural stigma on the availability of SGM-tailored programming at mental health treatment facilities was not observed.

**Table 3:** Orthogonal Reparameterization Results for the N-MHSS ADL(1, 1) Model

Variable	Short-Run Effect		Long-Run Effect	
	Med	95% CI	Med	95% CI
${\mathrm{SLP}}_{i,t}$	0.025	[$-$ 0.093, 0.141]	0.060	[$-$ 0.268, 0.340]
${\mathrm{SLP}}_{i,t-1}$	0.097	[$-$ 0.019, 0.210]	0.232	[$-$ 0.048, 0.571]
${\Delta \mathrm{GVFm}}_{i,t}$	$-$ 0.064	[$-$ 0.220, 0.088]	$-$ 0.152	[$-$ 0.632, 0.210]
${\Delta \mathrm{GVFm}}_{i,t-1}$	0.002	[$-$ 0.152, 0.160]	0.005	[$-$ 0.398, 0.417]

All values are percentages. Med is the median of the distribution of the posterior parameter estimates. 95% CI is the 95% credible interval. Boldface indicates a significant estimate. SLP indicates structural stigma. GVFm indicates mental health treatment facilities receiving government funding. Delta (Δ) is the first difference of GVFm. Subscript *i* indicates the state, subscript *t* indicates the year. See the Supplemental Materials for more details on the subscripts

## Discussion

This is the first study to examine whether structural stigma and government funding were associated with the availability of SGM-tailored programming at U.S. behavioral health facilities over time. As expected, reductions in structural stigma were positively associated with state-level increases in substance use treatment facilities offering SGM-tailored programming. A sensitivity analysis revealed that this effect remained significant after correcting for the over-reporting of SGM-tailored programming. The effect of structural stigma on the availability of SGM-tailored programming also remained significant after controlling for systematic ways that states differ that were not accounted for in the independent variables, such as the presence of SGM community enclaves in major cities. On average, in the model with the corrected estimate of SGM-tailored programming at substance use treatment facilities, reductions in structural stigma resulted in about two new SGM-tailored programs in the short term (i.e., within the year) and about 31 new SGM-tailored programs in the long term (i.e., over time) across the U.S. After controlling for pro-SGM policies enacted in the current year, one-year annual lagged reductions in structural stigma (i.e., pro-SGM policies enacted last year) also were associated with increases in the proportion of substance use treatment facilities offering SGM-tailored programming across U.S. states.

Taken together, these findings corroborate existing research on structural stigma as a determinant of SGM people’s health opportunities. Because SGM-tailored programming facilitates access to healthcare [56] and the current study found significant longitudinal associations between structural stigma and the availability of SGM-tailored programming, our findings support claims that reducing structural stigma increases access to behavioral health treatment specifically [30] and healthcare generally [28] among SGM people. Yet, more research is needed to understand the mechanisms, such as identity disclosure, driving the relationship between structural stigma and SGM-tailored programming. For instance, SGM people living in states with high structural stigma are less likely to disclose their sexual identity to healthcare providers compared to SGM people living in states with low structural stigma [29]. As such, structural stigma may prevent substance use treatment facilities from offering SGM-tailored programming by dissuading SGM people from coming out to their providers. In addition, providers in states with high structural stigma may not recognize the need for SGM-tailored programming. Alternatively, since structural stigma deters medical schools from providing adequate training in SGM health (i.e., not covering SGM health in classes) [27] and justifies interpersonal discrimination [21, 57], providers in states with high structural stigma may harbor discriminatory views towards SGM patients [58] which, in turn, might result in beliefs that SGM-tailored programming is unnecessary.

Findings from the sensitivity analysis indicate the importance of correcting for the over-reporting of SGM-tailored programming. Without correcting the dependent variable used in the N-SSATS models, the observed effect of structural stigma on the availability of SGM-tailored programming at substance use treatment facilities ballooned, and government funding became a significant predictor of the availability of SGM-tailored programming. This finding raises two concerns. First, there lacks clarity on what “SGM-tailored programming” means to participants in the N-SSATS survey that has persisted for over 15 years [8, 55] and leads to inflated estimates of the availability of this efficacious treatment modality. Because the inflated estimates changed the interpretation and meaning of our results (i.e., effect size, statistical significance), improving operationalization of SGM-tailored programming in the N-SSATS is needed. We recommend greater question clarity in how SAMHSA inquiries about SGM-tailored programming with the N-SSATS, possibly using the rating criteria from SGM experts [8, 55] as examples in the question.

Second, the response bias in the N-SSATS data (i.e., 70.8%–82.6%) is indicative of measurement error so substantial that the N-SSATS survey item is not a reliable estimator of SGM-tailored programming at substance use treatment facilities.^1^ We addressed this measurement error issue by reporting both the corrected and uncorrected estimate of SGM-tailored programming. Nonetheless, the substantial measurement error raises questions about existing research using the N-SSATS data to investigate SGM-tailored programming. Although existing studies use robust analytic techniques [11, 18, 19], the substantial response bias in how facilities report SGM-tailored programming [8, 55] could alter the substantive claims generated in these studies. Until specificity and clarity of the N-SSATS definition of SGM-tailored programming improves, caution is warranted when interpreting results from studies using the N-SSATS data. Because the N-SSATS is the largest survey of substance use facilities in the U.S., avoiding use of the N-SSATS data due to measurement error would exacerbate the problem of little existing literature on substance use treatment barriers and facilitators among SGM people. Therefore, at a minimum, we recommend that future studies report both the corrected and uncorrected estimate of SGM-tailored programming to increase the accuracy of claims drawn from the N-SSATS data and to improve generalizability of findings from the N-SSATS data.

Given that over-reporting altered the magnitude and significance of the effects in the model using the N-SSATS data, the finding that neither structural stigma nor government funding were significant predictors of SGM-tailored programming at mental health treatment facilities could be attributed to response bias. Thus, researchers need to verify whether the pattern of over-reporting in the N-SSATS data is also present in the N-MHSS data. Aside from the potential effect of over-reporting, the lack of significant relationships using the N-MHSS data may reflect the inconsistent annual reporting of mental health treatment facilities offering SGM-tailored programming [10]. That is, at mental health treatment facilities, SGM-tailored programming decreased from 2014 to 2016, but increased from 2017 to 2019, potentially reflecting a quadratic phenomenon, not a linear one. Non-linear modeling techniques (e.g., polynomial terms) may be needed to detect a relationship between SGM-tailored programming at mental health treatment facilities and structural determinants. Moreover, future research should investigate how and why SGM-tailored programming varies annually at mental health treatment facilities but exhibits a positive annual trend at substance use treatment facilities.

Two considerations might explain why government funding was not a significant predictor in either model. First, as shown in Table 1, the proportion of facilities receiving government funding remained, on average, somewhat constant across years, which might suggest that non-partisan factors unrelated to SGM healthcare are driving funding allocations for behavioral health facilities [34]. For instance, federal, state, and local funding for behavioral health might reflect policy efforts to reduce the disease burden of untreated mental illness, which is among the highest of all diseases [59]. Second, because the government funding variable includes grants [16, 17] and grants change over time based on awardees, funding priorities, and availability, facilities may receive government funding in one year but not in a subsequent period. Because the N-SSATS and N-MHSS data do not provide linking variables [11], it was not possible to track facility-level funding between periods. In other words, although there was no evidence for a longitudinal relationship between government funding and SGM-tailored programming at the state level, a longitudinal association may exist at the facility level.

### Implications for public health policy

Because reductions in structural stigma exhibited a significant, positive relationship with increases in SGM-tailored programming at substance use treatment facilities, and given that substance use disproportionately affects SGM people relative to the general population [1, 2], policymakers, advocates, and other stakeholders should focus on legislating state-level, protective SGM policies to promote health equity. According to the most recent pro-SGM policy map [60], to maximize the effect between policy and SGM-tailored programming availability observed in this paper, stakeholders should focus on reducing structural stigma (1) in states with negative-to-low overall policy scores (i.e., states characterized as “high priority” for achieving basic equality by the Human Rights Campaign [61]); (2) related to gender identity because, relative to sexual orientation (*n* = 5), there are many more states (*n* = 17) with deleterious policies towards transgender and nonbinary people, and those negative policies are increasing [62–64]; and (3) by prioritizing policies with wider support (e.g., non-discrimination, healthcare) over policies with stronger resistance (e.g., religious exemption, parental adoption). Public health officials can assist by educating policymakers on SGM behavioral health disparities [1, 7], advocating for the importance of SGM-tailored programming for reducing the SGM health burden [9], and explaining the demonstrated relationship between state-level policies and the availability of SGM-tailored programming at substance use treatment facilities.

### Limitations and future directions

Several limitations should be considered while interpreting this study’s findings. First, although we build a case for prospective causality by using panel analysis, this study does not establish causal inference because the significant association between structural stigma and the availability of SGM-tailored programming at substance use treatment facilities could be the result of unmeasured, confounding variables. We minimized this risk by introducing control variables for annual trending and state-level heterogeneity.

Second, the measurement error in the N-SSATS likely generated information bias. By correcting the dependent variable in the N-SSATS data (i.e., the percentage of substance use treatment facilities offering SGM-tailored programming) and comparing the results to a model whose results used an uncorrected dependent variable, we attempted to address this information bias. However, data we used to correct for over-reporting were from the 2018 N-SSATS data set and collected in 2020 [55], but the correction was applied retroactively and prospectively. It is possible that the measurement error changed significantly each year. Thus, our approach, while the first to attempt to account for this measurement error and information bias, inadequately addressed possible annual variation in measurement error. Given that only one other study has examined over-reporting and measurement error in the N-SSATS [8], more research is required to understand how over-reporting affects estimates of SGM-tailored programming in the U.S.

Third, although Ji’s [55] method of verifying the actual number of SGM-tailored programs reported in the N-SSATS entailed many strengths (e.g., multiple calls to all substance use treatment facilities stating they offer SGM-tailored programming, exceptional interrater reliability in their coding process), this method is not without limitations. For example, it is possible that the employees who answered the phone calls were unaware of specific programs offered by the facility (e.g., a new staff member).^2^ As Ji [55] notes in their limitations, there was no control group in their study design, so it is possible that Ji reached facilities whose staff members were more or less knowledgeable than staff members at other substance use treatment facilities. Importantly, Ji’s methods occurred during the COVID-19 pandemic and, as they note in their limitations, some facilities were closed or had reduced hours. Consequently, selection bias may have impacted Ji’s [55] estimates of the actual number of substance use treatment facilities offering SGM-tailored programming. Although there are limitations to the correction procedure we used in the sensitivity analysis, it is notable that Ji’s results mirror a previous study using similar procedures [8].

Fourth, this study aggregated data on SGM-tailored programming to the state level which, although useful from a panel analysis perspective, may have masked important multilevel factors (e.g., availability of local SGM community organizations at the county level, pro-SGM voting history of state legislators) that might have been detected in an approach like hierarchical linear modeling. Importantly, although a multilevel model to predict the facility-level probability of offering SGM-tailored programming may have yielded superior results because we could have accounted for the multilevel structure of these data (e.g., facilities nested in counties nested in states), it was not possible to use such a model because there are no linking identifiers in the N-SSATS and N-MHSS data sets. For example, there is no way to know whether Facility A in year 2015 is the same facility in 2016. Thus, aggregating variables to the state level was a necessary, if not preferable, analytic option.

Fifth, the N-SSATS aggregates government funding to include federal, state, and local sources, which limited the ability to focus on state-level government funding (which we did with the N-MHSS). Sixth, although selection bias is not a significant concern overall given that the responses rates for most years were > 80% for both the N-SSATS and the N-MHSS, the response rate varies by state each year [16, 17], so it is possible that selection bias affected results for specific states.

Finally, although orthogonal reparameterization was appropriate for our short panel, the main limitation was a restriction to a fixed-effects model [51]. That is, we were unable to model theoretically substantive, time-invariant variables even though they likely contribute to the availability of SGM-tailored programming. For example, baseline cultural acceptance of SGM people varies across states (e.g., states like New York and California have historically large, well-developed SGM communities) [36] but not across time, and might influence the relationship between structural stigma levels and the availability of SGM-tailored programming. We controlled for these time-invariant effects instead of modeling them. Future studies might consider examining time-invariant effects as predictors of SGM-tailored programming.

## Conclusion

This paper demonstrates the need for continued efforts aimed at reducing structural stigma nationwide, particularly in states where anti-SGM policies are maintained. Specifically, our results show that after controlling for over-reporting of SGM-tailored programming in substance use treatment facilities and systematic differences between states, there is a significant, positive effect on the creation of SGM-tailored programming when pro-SGM policies are enacted. Our results also highlight the need for clarifying the definition of SGM-tailored programming to correct for the over-reporting in the N-SSATS and, potentially, the N-MHSS. Finally, our results highlight the need for public health officials to educate lawmakers on the relationship between structural stigma and healthcare opportunities specific to SGM people in states where anti-SGM policies exist. In sum, these results highlight the need for legislation protecting SGM people at the federal level. Since the start of 2022, several anti-SGM related healthcare bills have been passed in states such as Alabama, Arizona, Florida, and Texas [62–64]. Given the deleterious consequences of structural stigma [39], benefits of SGM-tailored programming [8, 9], and the link between structural stigma and SGM-tailored programming, these bills represent a major obstacle to providing SGM people with high-quality and needed behavioral health services.

## Supplementary Information

**Additional file 1.** Supplemental Materials.

## Data Availability

The datasets and analytic scripts generated during the current study are available in the GitHub repository, https://github.com/CJCascalheira/structural_stigma.

## Footnotes

1 Thank you to the anonymous reviewer for emphasizing the point about measurement error.

2 Thank you to the anonymous reviewer who raised this possibility.
