# Supplementary material for: Structural determinants of tailored behavioral health services for sexual and gender minorities in the United States, 2010 to 2020: a panel analysis
Source: BMC Public Health. 2022 Oct 12;22:1908. doi: 10.1186/s12889-022-14315-1 (PMC9556150; doi:10.1186/s12889-022-14315-1)
Supplement: Supplementary file 1 — Additional file 1. Supplemental Materials. [file 12889_2022_14315_MOESM1_ESM.docx]

**Structural Determinants of Tailored Behavioral Health Services for Sexual and Gender Minorities in the United States, 2010 to 2020: A Panel Analysis**

**Supplemental Materials**

**Contents**

[Operationalization of Variables 2](#_Toc113353012)

[National Survey of Substance Abuse Treatment Services (N-SSATS; SAMHSA, 2021) 2](#_Toc113353013)

[National Mental Health Services Survey (N-MHSS; SAMHSA, 2020) 2](#_Toc113353014)

[Structural Stigma 2](#_Toc113353015)

[Response Rates 3](#_Toc113353016)

[Descriptive Statistics by Year 5](#_Toc113353017)

[Table 1 6](#_Toc113353018)

[Table 2 6](#_Toc113353019)

[Table 3 6](#_Toc113353020)

[Table 4 7](#_Toc113353021)

[Table 5 7](#_Toc113353022)

[Table 6 8](#_Toc113353023)

[N-SSATS Panel Data Assumption Tests 9](#_Toc113353024)

[Serial Correlation Tests 10](#_Toc113353025)

[Unit Root Tests 12](#_Toc113353026)

[Table 7 12](#_Toc113353027)

[Heteroskedasticity Test 14](#_Toc113353028)

[Balanced Panels 15](#_Toc113353029)

[N-SSATS Data: Comparison to the Ordinary Least Squares Estimator 16](#_Toc113353030)

[Table 8 17](#_Toc113353031)

[Table 9 17](#_Toc113353032)

[N-MHSS Panel Data Assumption Tests 17](#_Toc113353033)

[Serial Correlation Tests 19](#_Toc113353034)

[Unit Root Tests 20](#_Toc113353035)

[Table 10 20](#_Toc113353036)

[Heteroskedasticity Test 22](#_Toc113353037)

[Balanced Panels 23](#_Toc113353038)

[N-MHSS Data: Comparison to the Ordinary Least Squares Estimator 24](#_Toc113353039)

[Table 11 24](#_Toc113353040)

[Final Analytic Models 25](#_Toc113353041)

[References 28](#_Toc113353042)

# Operationalization of Variables

Variables were transformed into counts and then transformed into percentages because the number of behavioral health facilities across states varied substantially (e.g., in 2020, there were 1,734 substance use treatment facilities in California but only 49 in Delaware).

## National Survey of Substance Abuse Treatment Services (N-SSATS; SAMHSA, 2021)

- STATE = state where facility is located
- SRVC62 = facility offers sexual and gender minority (SGM) -tailored programming
- EARMARK = facility receives any federal, state, county, or local funds

## National Mental Health Services Survey (N-MHSS; SAMHSA, 2020)

- LST = state where facility is located
- SRVC62 = facility offers SGM-tailored programming
- FUNDOTHSTATE, FUNDLOCALGOV, FUNDSMHA, FUNDSTATEWELFARE, FUNDSTATEJUV, FUNDSTATEEDUC = types of government funding offered by the facility

## Structural Stigma

Structural stigma (i.e., state-level supportive SGM policies) was assessed with the Movement Advancement Project (MAP) [3]. MAP represents an index on the degree to which a state enacts supportive SGM policies and has been used in previous work [4]–[6]. Specifically, MAP tracks legislation relevant to SGM civil rights, such as anti-discrimination or adoption laws, and rates the degree to which states enact such laws. For instance, a state receives a higher anti-discrimination rating (i.e., a more supportive SGM rating) if it forbids discrimination based on sexual orientation and gender identity, a lower rating if it forbids discrimination based on sexual orientation only, and the lowest rating if it enacts no protections for SGM people. MAP policy data for 2010 to 2020 were scraped (i.e., downloaded from HTML code) from the Internet Archive using the Wayback Machine (for example code, see [7]).

Given that MAP altered their policy tracking strategy over time in response to emergent legislative initiatives, the total policy score for each year was transformed using the percent of maximum possible (“POMP”) method [8], instead of *z*-standardization, to preserve the distribution and covariance matrix of the transformed variables [9]. POMP linearly transforms each score to a percentage of the maximum score, thus the interpretability of sample and inference statistics (e.g., mean-level change across time points, regression coefficients) remains conventionally meaningful [8]. POMP scores are given by

$$\frac{\text{X}_{i}-\text{Min}}{\text{Max}-\text{Min}}\text{×100}$$

where $\text{X}_{i}$ is the observed value, Min is the minimum of each policy score within time points, and Max is the theoretical maximum of the policy score in 2020 (i.e., 38.5) [3] because the “max score used for the transformation must be the same values used to transform each time of measurement […] in order to see any longitudinal changes on the construct” [10, p. 19].

# Response Rates

Based on the annual reports of each survey, the average response rate (*M* = 85.72%) for the N-MHSS was 74.9% in 2014, 80.4% in 2015, 91% in 2016, 87% in 2017, 90% in 2018, and 91% in 2019; the response rate (*M* = 92%) for the N-SSATS was not reported in 2010 because there was no annual report, was 94% in 2011, 93% in 2012, 94% in 2013, 94% in 2014, 92% in 2015, 91% in 2016, 89% in 2017, 92% in 2018, 91% in 2019, and 90% in 2020. Response rates in the majority of years for both the N-MHSS and N-SSATS exceeded the 80% threshold typically deemed to reduce the chances of selection bias [42].

# Descriptive Statistics by Year

Tables 1 through 6 show the descriptive statistics of the key variables for each year, with Tables 1, 2, and 4 presenting data from the N-SSATS [1] and Tables 5 and 6 presenting data from the N-MHSS [2]. State-level data on legislative polices relevant to the rights and wellbeing of SGM people are tracked and published by the Movement Advancement Project [3]; these data are presented in Table 3. The variables for the N-SSATS-based data are:

- $\text{SGMP}_{i,t}$ = dependent variable; the percentage of substance use treatment facilities offering SGM-tailored programs within state *i* in year *t*;
- ${\text{SGMP}\text{*}}_{i,t}$ = dependent variable; the MASKED FOR REVIEW corrected percentage of substance use treatment facilities offering SGM-tailored programs within state *i* in year *t*;

- $\text{SLP}_{i,t}$ = the rating of state-level supportive SGM policies for state *i* in year *t*; and
- $\text{GVF}_{i,t}$ = the percentage of substance use treatment facilities within state *i* receiving government funding in year *t*.

The variables for the N-MHSS-based data are:

- ${\text{SGMP}\text{m}}_{i,t}$ = dependent variable; the percentage of mental health treatment facilities offering SGM-tailored programs within state *i* in year *t*;
- $\text{SLP}_{i,t}$ = the rating of state-level supportive SGM policies for state *i* in year *t*; and
- ${\text{GVF}\text{m}}_{i,t}$ = the percentage of mental health treatment facilities within state *i* receiving government funding in year *t*.

### Table 1

*Descriptive Statistics of N-SSATS Variable: Percentage of Substance Use Treatment Facilities Offering SGM-Tailored Programs in State (SGMP)*

| Year | M | SD | Median | Min | Max | Skew | Kurtosis |
| --- | --- | --- | --- | --- | --- | --- | --- |
| 2010 | 5.01 | 2.48 | 4.42 | 0.80 | 10.27 | 0.55 | $-$0.67 |
| 2011 | 4.22 | 2.39 | 3.85 | 0 | 9.88 | 0.24 | $-$0.76 |
| 2012 | 5.56 | 2.75 | 4.92 | 1.29 | 15.97 | 1.24 | 2.57 |
| 2013 | 10.54 | 4.47 | 10.00 | 2.27 | 21.47 | 0.37 | $-$0.51 |
| 2014 | 15.09 | 5.34 | 15.03 | 5.92 | 30.77 | 0.68 | 0.51 |
| 2015 | 15.79 | 5.30 | 16.29 | 5.17 | 26.48 | 0.08 | $-$0.8 |
| 2016 | 16.31 | 5.99 | 15.19 | 6.25 | 27.92 | 0.21 | $-$1.07 |
| 2017 | 17.12 | 6.53 | 16.67 | 4.48 | 36.36 | 0.40 | $-$0.09 |
| 2018 | 18.45 | 6.55 | 17.32 | 7.02 | 33.33 | 0.34 | $-$0.88 |
| 2019 | 20.57 | 6.61 | 20.08 | 8.24 | 36.75 | 0.38 | $-$0.67 |
| 2020 | 22.14 | 7.47 | 21.19 | 4.60 | 40.37 | 0.19 | $-$0.42 |

### Table 2

*Descriptive Statistics of N-SSATS Variable: Corrected Percentage of Substance Use Treatment Facilities Offering SGM-Tailored Programs in State (SGMP*)*

| Year | M | SD | Median | Min | Max | Skew | Kurtosis |
| --- | --- | --- | --- | --- | --- | --- | --- |
| 2010 | 0.61 | 0.58 | 0.44 | 0 | 3.17 | 1.85 | 5.33 |
| 2011 | 0.52 | 0.51 | 0.41 | 0 | 2.38 | 1.32 | 1.90 |
| 2012 | 0.63 | 0.51 | 0.49 | 0 | 2.33 | 0.98 | 0.63 |
| 2013 | 1.19 | 0.88 | 1.04 | 0 | 3.25 | 0.44 | $-$0.94 |
| 2014 | 1.74 | 1.24 | 1.48 | 0 | 4.56 | 0.43 | $-$0.85 |
| 2015 | 1.79 | 1.21 | 1.69 | 0 | 4.36 | 0.22 | $-$1.05 |
| 2016 | 1.82 | 1.27 | 1.70 | 0 | 4.74 | 0.43 | $-$0.69 |
| 2017 | 1.96 | 1.39 | 1.84 | 0 | 5.79 | 0.49 | $-$0.41 |
| 2018 | 2.12 | 1.60 | 1.83 | 0 | 6.24 | 0.65 | $-$0.29 |
| 2019 | 2.32 | 1.63 | 1.92 | 0 | 5.83 | 0.47 | $-$0.82 |
| 2020 | 2.40 | 1.65 | 2.14 | 0 | 6.42 | 0.38 | $-$0.76 |

*Note.* Figures from the MASKED FOR REVIEW correction; for an explanation, see section on N-SSATS serial correlation tests.

### Table 3

*Descriptive Statistics of N-SSATS and N-MHSS Variable: State-Level Supportive SGM Policies (SLP)*

| Year | M | SD | Median | Min | Max | Skew | Kurtosis |
| --- | --- | --- | --- | --- | --- | --- | --- |
| 2010 | 20.67 | 12.86 | 18.07 | 0 | 43.37 | 0.09 | $-$1.40 |
| 2011 | 29.35 | 21.35 | 16.87 | 0 | 62.65 | 0.38 | $-$1.60 |
| 2012 | 35.26 | 28.69 | 20.69 | 0 | 82.76 | 0.41 | $-$1.59 |
| 2013 | 42.62 | 33.77 | 24.73 | 0 | 98.92 | 0.37 | $-$1.68 |
| 2014 | 42.53 | 35.67 | 25.29 | 0 | 98.85 | 0.24 | $-$1.71 |
| 2015 | 27.58 | 23.85 | 14.56 | 0 | 76.58 | 0.58 | $-$1.23 |
| 2016 | 28.00 | 25.74 | 13.96 | 0 | 78.57 | 0.60 | $-$1.29 |
| 2017 | 32.43 | 27.03 | 19.51 | 0 | 84.15 | 0.50 | $-$1.40 |
| 2018 | 35.51 | 28.34 | 23.78 | 0 | 86.59 | 0.38 | $-$1.54 |
| 2019 | 39.35 | 31.16 | 26.83 | 0 | 93.90 | 0.35 | $-$1.58 |
| 2020 | 47.03 | 31.92 | 39.44 | 0 | 92.22 | 0.19 | $-$1.63 |

### Table 4

*Descriptive Statistics of N-SSATS Variable: Percentage of Substance Use Treatment Facilities Within a State Receiving Government Funding (GVF)*

| Year | M | SD | Median | Min | Max | Skew | Kurtosis |
| --- | --- | --- | --- | --- | --- | --- | --- |
| 2010 | 63.27 | 12.45 | 62.18 | 35.75 | 87.39 | 0.04 | $-$0.72 |
| 2011 | 62.87 | 11.94 | 62.94 | 36.62 | 89.08 | 0.18 | $-$0.59 |
| 2012 | 61.20 | 12.19 | 59.89 | 33.63 | 87.04 | 0.08 | $-$0.49 |
| 2013 | 60.63 | 12.90 | 59.54 | 32.43 | 86.18 | $-$0.10 | $-$0.56 |
| 2014 | 58.38 | 13.14 | 58.98 | 34.23 | 97.81 | 0.47 | 0.16 |
| 2015 | 57.99 | 12.91 | 55.98 | 31.25 | 88.76 | 0.37 | $-$0.28 |
| 2016 | 55.35 | 14.03 | 52.96 | 29.26 | 89.66 | 0.43 | $-$0.40 |
| 2017 | 56.60 | 13.33 | 55.65 | 34.93 | 87.5 | 0.36 | $-$0.66 |
| 2018 | 56.98 | 13.31 | 55.67 | 30.51 | 85.71 | 0.26 | $-$0.57 |
| 2019 | 55.92 | 13.29 | 55.07 | 23.90 | 85.63 | 0.05 | $-$0.40 |
| 2020 | 55.36 | 13.32 | 54.85 | 22.50 | 85.7 | 0.08 | $-$0.36 |

### Table 5

*Descriptive Statistics of N-MHSS Variables: Percentage of Mental Health Treatment Facilities Offering SGM-Tailored Programs in State (SGMPm)*

| Year | M | SD | Median | Min | Max | Skew | Kurtosis |
| --- | --- | --- | --- | --- | --- | --- | --- |
| 2014 | 22.89 | 5.38 | 22.51 | 9.95 | 33.90 | $-$0.05 | $-$0.25 |
| 2015 | 17.09 | 5.33 | 16.82 | 6.02 | 29.03 | 0.02 | $-$0.47 |
| 2016 | 12.40 | 4.73 | 12.78 | 3.23 | 24.14 | 0.09 | $-$0.44 |
| 2017 | 15.85 | 6.34 | 15.32 | 3.92 | 36.36 | 0.93 | 1.43 |
| 2018 | 17.84 | 5.29 | 17.48 | 6.12 | 32.16 | 0.27 | $-$0.24 |
| 2019 | 20.58 | 6.88 | 20.60 | 8.16 | 42.42 | 0.79 | 1.31 |

### Table 6

*Descriptive Statistics of N-MHSS Variables: Percentage of Mental Health Treatment Facilities Within a State Receiving Government Funding (GVFm)*

| Year | M | SD | Median | Min | Max | Skew | Kurtosis |
| --- | --- | --- | --- | --- | --- | --- | --- |
| 2014 | 86.5 | 6.92 | 88.08 | 68.18 | 98.18 | $-$0.80 | 0.22 |
| 2015 | 85.69 | 7.23 | 85.48 | 63.44 | 100.00 | $-$0.66 | 0.55 |
| 2016 | 85.66 | 6.96 | 85.74 | 63.10 | 98.04 | $-$0.78 | 0.93 |
| 2017 | 85.94 | 6.54 | 85.76 | 61.36 | 100.00 | $-$0.82 | 2.30 |
| 2018 | 85.57 | 6.74 | 86.71 | 57.69 | 97.92 | $-$1.29 | 3.94 |
| 2019 | 75.86 | 9.29 | 76.66 | 41.8 | 93.33 | $-$1.05 | 2.24 |

# N-SSATS Panel Data Assumption Tests

Similar to existing work [12], prior to presenting the final dynamic autoregressive distributed lag (ADL[1, 1]) model, the following assumptions were investigated for the panel data (i.e., longitudinal analyses): serial correlation tests, unit root tests, heteroskedasticity tests, and balanced panels. The original ADL(1, 1) model was in the form

$$\text{SGMP}_{i,t}={\rho\text{SGMP}}_{i,t-1}+{\beta_{1}\text{SLP}}_{i,t}+{\beta_{2}\text{SLP}}_{i,t-1}+{\beta_{3}\text{GVF}}_{i,t}+{\beta_{4}\text{GVF}}_{i,t-1}+\tau_{t}+ \eta_{i}+\varepsilon_{i, t}$$

where the dependent variable ($\text{SGMP}_{i,t}$) is the percentage of substance use treatment facilities offering SGM-tailored programs within state *i* in year *t*. Since the percentage of substance use treatment facilities offering SGM-tailored programs within a state is related to the percentage of programs in the previous year (average annual correlation = .945), a coefficient (*ρ*) on the lag of the dependent variable was estimated ($\text{SGMP}_{i,t-1}$) because the dependent variable is autoregressive [13]. $\text{SLP}_{i,t}$ represents the rating of state-level supportive SGM policies for state *i* in year *t* and in the previous year *t*–1 ($\text{SLP}_{i,t-1}$) while $\text{GVF}_{i,t}$ is the percentage of substance use treatment facilities within state *i* receiving government funding in year *t* ($\text{GVF}_{i,t-1}$ is one year before). A fixed-effects model was chosen to account for unobserved time-invariant characteristics and since no key variables were time-invariant. To control for unmodelled time-specific annual trending, a dummy variable for year ($\tau_{t}$) was added; $\eta_{i}$ was used to control for unobserved unit-specific effects and $\varepsilon_{i, t}$ is the error term [14].

## Serial Correlation Tests

Temporal (serial) autocorrelation was examined with the Arellano and Bond [15] $\text{m}_{\text{2}}$ statistic in R. The null hypothesis is that the residuals are white noise. Thus, there is evidence of serial correlation if the null hypothesis is rejected. When serial autocorrelation is present, one can (a) specify additional lags of the independent or dependent variables, (b) take the first difference of all variables on both sides of the equation, (c) specify additional variables that may have been omitted during the original model specification, (d) apply a transformation, or (e) compute robust standard errors [16], [17]. Since the $\text{m}_{\text{2}}$ statistic for the original ADL(1, 1) model was not significant ($\text{m}_{\text{2}}$ = 0.466, *p* = .641), there was no evidence of serial autocorrelation.

Based on work by Ji and Cochran [4], a correction to $\text{SGMP}_{i,t}$ was applied. Ji and Cochran [11] conducted structured telephone surveys with all facilities claiming to offer SGM-tailored programming. Given the robustness of their method, their data was used as the actual number of SGM-tailored programming per state. Given Cochran et al. [18] found a similar pattern of findings, we assumed that facilities overreported their SGM-tailored programming between 2010 and 2020. Therefore, a proportion was calculated to correct the dependent variable:

$$\frac{\text{confirmed number of facilities offering SGM-tailored programming}\text{ }}{\text{N-SSATS 2020 number of facilities claiming to offer SGM-tailored programming}}$$

such that each state’s number of SGM-tailored programs for years 2010 to 2020 were readjusted as the product of this proportion times $\text{SGMP}_{i,t}$. The new total number of facilities offering SGM-tailored programming was divided by the total number of facilities to yield the new, corrected percentage of facilities offering SGM-tailored programming. After applying the correction, serial autocorrelation remained absent ($\text{m}_{\text{2}}$ = 0.186, *p* = .852). Therefore, the original model was used in the main analysis:

$$\text{SGMP}_{i,t}={\rho\text{SGMP}}_{i,t-1}+{\beta_{1}\text{SLP}}_{i,t}+{\beta_{2}\text{SLP}}_{i,t-1}+{\beta_{3}\text{GVF}}_{i,t}+{\beta_{4}\text{GVF}}_{i,t-1}+\tau_{t}+ \eta_{i}+\varepsilon_{i, t}$$

## Unit Root Tests

Panel data should be stationary; but if both *x* and *y* are nonstationary, then either (a) the first difference of all nonstationary variables should be taken prior to estimation to remove the unit root or (b) the practitioner should test for a cointegrating relationship [19]. Unit root tests provide evidence for the stationarity assumption [19]. To determine whether the variables contained a unit root, the Fisher-type Philips-Perron unit-root test with 1 lag, demeaned data, and a time trend was applied to each variable in StataBE version 17 using the command xtunitroot fisher variable, demean trend pperron lags(1) [12]. The hypotheses are:

$H_{0}$: all panels contain a unit root; and

$H_{1}$: at least one panel is stationary.

All four test statistics produced by the test (i.e., inverse $\chi^{2}$, inverse normal, inverse logit transformation, and the modified inverse $\chi^{2}$) were in agreement for all variables. Thus, the inverse $\chi^{2}$ is reported in the table below. As shown in the table below, we failed to reject the null hypothesis for $\text{SLP}_{i,t}$ and $\text{SGMP}_{i,t}$ (corrected) but succeeded in rejecting the null hypothesis for $\text{GVF}_{i,t}$ and $\text{SGMP}_{i,t}$. In summary, all panels contain a unit root for $\text{SLP}_{i,t}$ and $\text{SGMP}_{i,t}$ (corrected) and at least one panel is stationary for $\text{GVF}_{i,t}$, and $\text{SGMP}_{i,t}$.

### Table 7

*Results from the Fisher-type Philips-Perron Unit-Root Test*

| Variable | Inverse $\chi^{2}$ | *p* value |
| --- | --- | --- |
| $\text{SGMP}_{i,t}$ | 131.3941 | .019 |
| $\text{SGMP}_{i,t}$ (corrected) | 91.1639 | .725 |
| $\text{SLP}_{i,t}$ | 111.3541 | .206 |
| $\text{GVF}_{i,t}$ | 181.0034 | < .001 |

Given that it is best practice to apply at least two unit-root tests when assessing the stationarity assumption (Andrew Q. Philips, personal communication), the Levin-Lin-Chu (LLC) unit-root test was conducted. The LLC unit-root test, which is considered a more stringent test of stationarity [12], was selected because the data are a short panel (*N* / *T* → 0, 50 / 10 = 5) and other unit-root tests assume *N* or *T* approaching infinity [19]. The hypotheses are:

$H_{0}$: all panels contain a unit root; and

$H_{1}$: all panels are stationary.

Results from the LLC unit-root test, with a trend and 1 lag, were significant for $\text{SGMP}_{i,t}$ (adjusted *t* = $-$17.79, p < .001), significant for $\text{SGMP}_{i,t}$ (corrected; adjusted *t* = $-$15.93, p < .001), significant for $\text{SLP}_{i,t}$ (adjusted *t* = $-$18.29, p < .001), and significant for $\text{GVF}_{i,t}$ (adjusted *t* = $-$16.81, p < .001). Therefore, the LLC unit-root test indicated that all panels for all variables are stationary. In light of the results from the unit-root tests and considering that (a) controlling for trending often promotes stationarity and (b) when *T* is small, “bias due to […] nonstationary is negligible” [13, p. 2], this short panel was assumed to be stationary for the ADL(1, 1) with $\text{SGMP}_{i,t}$ (corrected) as the dependent variable.

## Heteroskedasticity Test

Heteroskedasticity exists when the error term can be predicted from the explanatory variables, and most panel models assume the absence of heteroskedasticity [19]. The Breush-Pagan test for heteroskedasticity was executed in StataBE version 17. Using ordinary least squares (OLS) regression, Breush-Pagan test for heteroskedasticity examines the correlation between a model’s explanatory variables and its residuals. The null hypothesis is that the residuals are homoscedastic (i.e., the error is not predicted from the explanatory variables). Results yielded a Breush-Pagan statistic of 65.97 (*p* < .001), thus the null hypothesis was rejected and there was evidence for heteroscedasticity. Typically, the presence of heteroskedasticity warrants the use of robust standard errors [19]. However, orthogonal reparameterization (i.e., the current estimation method) is largely robust to heteroskedastic errors [13], so no corrections to the standard errors were specified. Indeed, standard errors are not calculated in orthogonal reparameterization [14].

## Balanced Panels

A balanced panel is one in which measurements on the same individuals are available across all years [19]. Only government funding data was missing for 2014 across all states, so values were replaced with expectation maximization using the R package *Amelia II* [20]. After imputation, there were no missing data for any states across any years and, thus, the panels were balanced.

# N-SSATS Data: Comparison to the Ordinary Least Squares Estimator

It is common practice to compare advanced models to OLS to, for instance, check for discrepancies, demonstrate how OLS produces biased estimates, and discuss the power of advanced models [19], [21], [22]. One particular function of this comparison is the robustness check [12], which allows the analyst to ascertain how “‘core’ regression coefficient estimates behave when the regression specification is modified in some way” [23, p. 1]. According to Lu and White [23], results from robustness checks typically imply that the interpretation of the regression coefficients is reliable. This step is not an assumption test and, thus, is not critical to the estimation of an ADL(1, 1) model using orthogonal reparameterization.

A dynamic fixed-effects OLS estimator was specified to compare the regression coefficients to the median posterior estimates from orthogonal reparameterization. The median posterior estimate of *ρ* was 0.935 with a 95% credible interval that did not include zero. In contrast, the coefficient on the lagged dependent variable in the OLS model was much smaller and non-significant (*β* = 0.062 [*SE* = 0.042], *p* = .137). The lagged dependent variable was included to account for autoregression in the dependent variable [13], so although there is a discrepancy between the two models, it does not bear upon the substantive short-run interpretations gleaned from the models. However, it will influence the long-run effects [19], [22]. In terms of the short-run effects, the remaining results from both models are presented in Table 8 and are largely consistent with one another. Only the short-run effect for $\text{SLP}_{i,t}$ was significant in both estimators; however, $\text{SLP}_{i,t-1}$ also was significant in the orthogonal reparameterization estimator. The magnitudes of the OLS coefficients were larger overall. The directionality of the median of the posterior estimates on $\text{SLP}_{i,t-1}$ and $\text{GVF}_{i,t-1}$ changed in OLS. In summary, there is evidence that the estimates are most robust for $\text{SLP}_{i,t}$.

### Table 8

*Short-Run Effects from Orthogonal Reparameterization Versus the OLS Estimator*

| Variable |  | Orthogonal Reparameterization | |  | OLS Fixed-Effects Estimator | | |
| --- | --- | --- | --- | --- | --- | --- | --- |
|  |  | Med | 95% CI |  | *β* | *SE* | *p* |
| $\text{SLP}_{i,t}$ |  | **0.007** | **[0.003, 0.012]** |  | **0.226** | **0.060** | **< .001** |
| $\text{SLP}_{i,t-1}$ |  | **0.004** | **[0.0004, 0.007]** |  | –0.066 | 0.046 | .156 |
| $\text{GVF}_{i,t}$ |  | $-$0.006 | [$-$0.013, 0.001] |  | 0.029 | 0.042 | .484 |
| $\text{GVF}_{i,t-1}$ |  | $-$0.0002 | [$-$0.006, 0.006] |  | –0.026 | 0.034 | .462 |

*Note*. All values are percentages. Med is the median of the distribution of the posterior parameter estimates. 95% CI is the 95% credible interval. SE is the standard errors. Boldface indicates significance.

Given the discrepant values of *ρ*, the long-run effects were notably different. The directionality changes did not differ from the short-run effects. However, as shown in Table 9, the magnitude of the long-run effect (long-run effect = $\frac{\beta}{1-\rho}$) was smaller for the median of posterior estimates than the equivalent OLS coefficients for $\text{SLP}_{i,t}$, $\text{SLP}_{i,t-1}$, and $\text{GVF}_{i,t-1}$, but larger for $\text{GVF}_{i,t}$.

### Table 9

*Long-Run Effects from Orthogonal Reparameterization Versus the OLS Estimator*

| Variable |  | Orthogonal Reparameterization |  | OLS Fixed-Effects Estimator |
| --- | --- | --- | --- | --- |
|  |  | Med |  | *β** |
| $\text{SLP}_{i,t}$ |  | **0.109** |  | **0.241** |
| $\text{SLP}_{i,t-1}$ |  | **0.058** |  | –0.070 |
| $\text{GVF}_{i,t}$ |  | –0.088 |  | 0.031 |
| $\text{GVF}_{i,t-1}$ |  | –0.004 |  | –0.027 |

*Note*. Med is the median of the distribution of the posterior parameter estimates (i.e., the long-run effect). *β** is the long-run effect of the OLS estimates. Boldface indicates significance.

# N-MHSS Panel Data Assumption Tests

The following assumptions also were investigated for the N-MHSS-based data: serial correlation tests, unit root tests, heteroskedasticity tests, and balanced panels. The original ADL(1, 1) model was in the form

$${\text{SGMP}\text{m}}_{i,t}={\rho\text{SGMP}\text{m}}_{i,t-1}+{\beta_{1}\text{SLP}}_{i,t}+{\beta_{2}\text{SLP}}_{i,t-1}+{\beta_{3}\text{GVF}\text{m}}_{i,t}+{\beta_{4}\text{GVF}\text{m}}_{i,t-1}$$

$$+\tau_{t}+ \eta_{i}+\varepsilon_{i, t}$$

where the dependent variable (${\text{SGMP}\text{m}}_{i,t}$) is the percentage of mental health facilities offering SGM-tailored programs within state *i* in year *t*; *ρ* is the coefficient on the lag of the dependent variable (${\text{SGMP}\text{m}}_{i,t-1}$). Again, the dependent variable is autoregressive [13]. $\text{SLP}_{i,t}$ represents the rating of state-level supportive SGM policies for state *i* in year *t* and in the previous year *t*–1 ($\text{SLP}_{i,t-1}$). ${\text{GVF}\text{m}}_{i,t}$ is the percentage of mental health treatment facilities within state *i* receiving government funding in year *t* (${\text{GVF}\text{m}}_{i,t-1}$ is one year before). Once again, a fixed-effects model was chosen to account for unobserved time-invariant characteristics and since no key variables were time-invariant. The other variables ($\tau_{t}$, $\eta_{i}$, $\varepsilon_{i, t}$) have equivalent meaning to the variables in the N-SSATS model presented above [14].

## Serial Correlation Tests

The Arellano and Bond [15] $\text{m}_{\text{2}}$ statistic was calculated in R to provide evidence for serial correlation. The $\text{m}_{\text{2}}$ statistic for the original ADL(1, 1) model was not significant ($\text{m}_{\text{2}}$ = $-$0.531, *p* = .595) and, thus, there was no evidence of serial autocorrelation. Attempts to resolve the issue of serial autocorrelation were made. Therefore, the following model was used in the main analysis:

$${\text{SGMP}\text{m}}_{i,t}={\rho\text{SGMP}\text{m}}_{i,t-1}+{\beta_{1}\text{SLP}}_{i,t}+{\beta_{2}\text{SLP}}_{i,t-1}+{\beta_{3}\text{GVF}\text{m}}_{i,t}+{\beta_{4}\text{GVF}\text{m}}_{i,t-1}+\tau_{t}+ \eta_{i}+\varepsilon_{i, t}$$

## Unit Root Tests

Once again, to determine whether the variables contained a unit root, the Fisher-type Philips-Perron unit-root test with 1 lag, demeaned data, and a time trend was applied to each variable in StataBE version 17. Rejecting the null hypothesis indicates that at least one panel is stationary. All four test statistics produced by the test (i.e., inverse $\chi^{2}$, inverse normal, inverse logit transformation, and the modified inverse $\chi^{2}$) were in agreement for all variables except $\text{GVF}_{i,t}$. Thus, the inverse $\chi^{2}$ is reported in the table below. For $\text{GVF}_{i,t}$, the inverse normal statistic was not significant (0.23, *p* = .593), but the other statistics produced by the Fisher-type Philips-Perron unit-root test were significant, suggesting that at least one panel is stationary. We rejected the null hypothesis for $\text{SGMP}_{i,t}$ and $\text{SLP}_{i,t}$, which provided evidence that at least one panel is stationary for each variable. Overall, the Fisher-type Philips-Perron unit-root test provided evidence that unit roots are not an issue.

### Table 10

*Results from the Fisher-type Philips-Perron Unit-Root Test*

| Variable | Inverse $\chi^{2}$ | *p* value |
| --- | --- | --- |
| $\text{SGMP}_{i,t}$ | 633.7059 | < .001 |
| $\text{SLP}_{i,t}$ | 2,138.9617 | < .001 |
| $\text{GVF}_{i,t}$ | 214.4861 | < .001 |

Applying at least two unit root tests is best practice. Once again, the LLC unit-root test was selected because the data are a short panel (*N* / *T* → 0, 50 / 6 = 8.33) and other unit-root tests assume *N* or *T* approaching infinity [19]. Rejecting the null hypothesis indicates that all panels are stationary. Results from the LLC unit-root test, with 1 lag, were significant for ${\text{SGMP}\text{m}}_{i,t}$ (adjusted *t* = $-$13.51, p < .001) and significant for $\text{SLP}_{i,t}$ (adjusted *t* = $-$11.19, p < .001), but not significant for ${\text{GVF}\text{m}}_{i,t}$ (adjusted *t* = 11.90, p = 1). Therefore, the LLC unit-root test indicated that all panels for are stationary for ${\text{SGMP}\text{m}}_{i,t}$ and $\text{SLP}_{i,t}$, but non-stationary for ${\text{GVF}\text{m}}_{i,t}$.

Therefore, the first difference of ${\text{GVF}\text{m}}_{i,t}$ was taken to remove the unit root [19]. Given that (a) the LLC unit-root test requires strongly balanced data and (b) the first difference operation generates a missing value in the first row of the data set (i.e., a lag of the first observation is missing since no data is preset), the Fisher-type Philips-Perron unit-root test was executed to determine if the first difference of ${\text{GVF}\text{m}}_{i,t}$ was stationary. All four tests were significant (inverse $\chi^{2}$ = 373.45, *p* < .001; inverse normal = $-$2.22, *p* < .001; inverse logit transformation = $-$8.66, *p* < .001; and the modified inverse $\chi^{2}$ = 19.34, *p* < .001), thereby providing evidence that the first difference of ${\text{GVF}\text{m}}_{i,t}$ was stationary. Importantly, taking the first difference of [19] ${\text{GVF}\text{m}}_{i,t}$ did not introduce serial autocorrelation into the ADL(1, 1) model ($\text{m}_{\text{2}}$ = $-$0.388, *p* = .678). Therefore, the new model used in the main analysis to account for the first difference ($\text{Δ}$) of ${\text{GVF}\text{m}}_{i,t}$ was:

$${\text{SGMP}\text{m}}_{i,t}={\rho\text{SGMP}\text{m}}_{i,t-1}+{\beta_{1}\text{SLP}}_{i,t}+{\beta_{2}\text{SLP}}_{i,t-1}+{\beta_{3}\text{Δ}\text{GVF}\text{m}}_{i,t}+{\beta_{4}\text{Δ}\text{GVF}\text{m}}_{i,t-1}$$

$$+\tau_{t}+ \eta_{i}+\varepsilon_{i, t}$$

## Heteroskedasticity Test

To assess heteroskedasticity, the Breush-Pagan test for heteroskedasticity was executed in StataBE version 17. Using ordinary least squares (OLS) regression, Breush-Pagan test for heteroskedasticity examines the correlation between a model’s explanatory variables and its residuals. The null hypothesis is that the residuals are homoscedastic (i.e., the error is not predicted from the explanatory variables). Results yielded a Breush-Pagan statistic of 0.58 (*p* = .447), thus the null hypothesis was not rejected and there was evidence for homoscedasticity.

## Balanced Panels

None of the data were missing for the N-MHSS-based panel—there were no gap years and each variable had observations for every year. Thus, the data were balanced.

# N-MHSS Data: Comparison to the Ordinary Least Squares Estimator

Once again, a dynamic fixed-effects OLS estimator was specified to compare the regression coefficients to the median posterior estimates from orthogonal reparameterization. The LREs were not calculated because both estimators yielded non-significant estimates. The median posterior estimate of *ρ* was 0.577 with a 95% credible interval that did not include zero. In contrast, the coefficient on the lagged dependent variable in the OLS model was much smaller and non-significant (*β* = $-$0.0641 [*SE* = 0.063], *p* = .312). In terms of the short-run effects, the remaining results from both models are presented in Table 11 and are largely consistent with one another. In summary, neither estimator yielded significant estimates.

### Table 11

*Short-Run Effects from Orthogonal Reparameterization Versus the OLS Estimator*

| Variable |  | Orthogonal Reparameterization | |  | OLS Fixed-Effects Estimator | | |
| --- | --- | --- | --- | --- | --- | --- | --- |
|  |  | Med | 95% CI |  | *β* | *SE* | *p* |
| $\text{SLP}_{i,t}$ |  | 0.025 | [$-$0.093, 0.141] |  | –0.002 | 0.018 | .99 |
| $\text{SLP}_{i,t-1}$ |  | 0.097 | [$-$0.019, 0.210] |  | 0.049 | 0.014 | .73 |
| $\Delta\text{GVF}_{i,t}$ |  | $-$0.064 | [$-$0.220, 0.088] |  | 0.060 | 0.027 | .53 |
| ${\text{Δ}\text{GVF}}_{i,t-1}$ |  | 0.002 | [$-$0.152, 0.160] |  | 0.068 | 0.023 | .48 |

*Note*. Med is the median of the distribution of the posterior parameter estimates. 95% CI is the 95% credible interval. SE is the standard errors.

# Final Analytic Models

Two dynamic panel, autoregressive-distributed lag (ADL[1, 1]) regression models with fixed effects were estimated [40] to identify the relationship between the percentage of behavioral health facilities offering SGM-tailored programming within a state, the rating of state-level supportive SGM policies (i.e., structural stigma), and the percentage of behavioral health facilities within a state receiving government funding. The final ADL(1, 1) models were created after examining assumptions, which are presented in the Supplemental Materials (p. 7–22). For substance use treatment facilities (i.e., N-SSATS data), the ADL(1, 1) model was:

$$\text{SGMP}_{i,t}={\rho\text{SGMP}}_{i,t-1}+{\beta_{1}\text{SLP}}_{i,t}+{\beta_{2}\text{SLP}}_{i,t-1}+{\beta_{3}\text{GVF}}_{i,t}+{\beta_{4}\text{GVF}}_{i,t-1}+\tau_{t}+ \eta_{i}+\varepsilon_{i, t}$$

where the dependent variable ($\text{SGMP}_{i,t}$) is the percentage of substance use treatment facilities offering SGM-tailored programs within state *i* in year *t*. Since the percentage of substance use treatment facilities offering SGM-tailored programs within a state is related to the percentage of programs in the previous year (average annual correlation = .962), a coefficient (*ρ*) on the lag of the dependent variable was estimated ($\text{SGMP}_{i,t-1}$) because the dependent variable is autoregressive [52]. $\text{SLP}_{i,t}$ represents the rating of state-level supportive SGM policies for state *i* in year *t* and in the previous year *t*–1 ($\text{SLP}_{i,t-1}$) while $\text{GVF}_{i,t}$ is the percentage of substance use treatment facilities within state *i* receiving government funding in year *t* ($\text{GVF}_{i,t-1}$ is one year before). A fixed-effects model was chosen to account for unobserved time-invariant characteristics and since no key variables were time-invariant. To control for unmodelled time-specific annual trending, a dummy variable for year ($\tau_{t}$) was added; $\eta_{i}$ was used to control for unobserved unit-specific effects and $\varepsilon_{i, t}$ is the error term [53].

For mental health treatment facilities (i.e., N-MHSS data), the ADL(1, 1) model was:

$${\text{SGMP}\text{m}}_{i,t}={\rho\text{SGMP}\text{m}}_{i,t-1}+{\beta_{1}\text{SLP}}_{i,t}+{\beta_{2}\text{SLP}}_{i,t-1}+{\beta_{3}\text{Δ}\text{GVF}\text{m}}_{i,t}+{\beta_{4}\text{Δ}\text{GVF}\text{m}}_{i,t-1}$$

$$+\tau_{t}+ \eta_{i}+\varepsilon_{i, t}$$

where the dependent variable (${\text{SGMP}\text{m}}_{i,t}$) is the percentage of mental health facilities offering SGM-tailored programs within state *i* in year *t*; *ρ* is the coefficient on the lag of the dependent variable (${\text{SGMP}\text{m}}_{i,t-1}$). Again, the dependent variable is autoregressive [52]. $\text{SLP}_{i,t}$ represents the rating of state-level supportive SGM policies for state *i* in year *t* and in the previous year *t*–1 ($\text{SLP}_{i,t-1}$). ${\text{Δ}\text{GVF}\text{m}}_{i,t}$ is the first difference of the percentage of mental health treatment facilities within state *i* receiving government funding in year *t* (${\text{Δ}\text{GVF}\text{m}}_{i,t-1}$ is one year before). The first difference, which is given by ${{\text{Δ}\text{x}}_{i,t} {\text{= }\text{x}}_{i,t}-\text{x}}_{i,t-1}$, was used to eliminate a unit root (see Supplemental Materials, p. 18) [40]. Once again, a fixed-effects model was chosen to account for unobserved time-invariant characteristics and since no key variables were time-invariant. The other variables ($\tau_{t}$, $\eta_{i}$, $\varepsilon_{i, t}$) have equivalent meaning to the variables in the N-SSATS-based model presented above.

Since the ADL(1, 1) models were fitted to short panels (i.e., N-SSATS: total number of states [$\text{N}_{\text{N-SSATS}}$] = 50, total number of time points [$\text{T}_{\text{N-SSATS}}$] = 10; N-MHSS: $\text{N}_{\text{N-MHSS}}$ = 50, $\text{T}_{\text{N-MHSS}}$ = 6), the ordinary least squares (OLS), the generalized least squares random-effects, and the OLS fixed-effects estimators are biased and inconsistent [40], [52], [54]. Typical solutions to this problem include the use of instrumental variables, likelihood-based estimators, and the generalized method of moments estimator [40]. However, an alternative is the orthogonal reparameterization (OPM) estimator [55], which is a Bayesian method. Pickup and Hopkins [52] conducted Monte Carlo simulations to show that, compared to the other estimators designed for short panels, the OPM estimator has the best properties for *N* $\leq$ 100 (e.g., low bias on the long-run effects [LREs], greater efficiency across coefficients) and is robust to violations of distributional assumptions. Therefore, OPM was used to estimate the models. *OrthoPanels* yields the short-run effect of the posterior parameters, which then is used to calculate the LRE with $\beta/1-\rho$ [53].

# References

[1] SAMHSA, “National Survey Of Substance Abuse Treatment Services (N-SSATS): 2020 - data on substance abuse treatment facilities,” *Substance Abuse and Mental Health Services Administration*, 2021. https://www.samhsa.gov/data/sites/default/files/reports/rpt35313/2020_NSSATS_FINAL.pdf

[2] SAMHSA, “National Mental Health Services Survey (N-MHSS): 2019 - data on mental health treatment facilities,” *Substance Abuse and Mental Health Services Administration*, 2020. https://wwwdasis.samhsa.gov/dasis2/nmhss/2019-NMHSS-R.pdf

[3] Movement Advancement Project, “Mapping LGBTQ equality: 2010 to 2020,” Feb. 2020. https://www.lgbtmap.org/2020-tally-report

[4] A. Flentje *et al.*, “Minority stress, structural stigma, and physical health among sexual and gender minority individuals: Examining the relative strength of the relationships,” *Annals of Behavioral Medicine*, p. kaab051, 2021, doi: 10.1093/abm/kaab051.

[5] N. K. Tran, M. L. Hatzenbuehler, and N. D. Goldstein, “Potential relationship between HIV criminalization and structural stigma related to sexual orientation in the United States,” *JAIDS Journal of Acquired Immune Deficiency Syndromes*, vol. 80, no. 5, pp. e106–e108, 2019, doi: 10.1097/QAI.0000000000001961.

[6] R. J. Watson, J. N. Fish, W. Denary, A. Caba, C. Cunningham, and L. A. Eaton, “LGBTQ state policies: A lever for reducing SGM youth substance use and bullying,” *Drug and Alcohol Dependence*, vol. 221, p. 108659, 2021, doi: 10.1016/j.drugalcdep.2021.108659.

[7] C. J. Cascalheira, “Structural stigma over time,” *Github*, Dec. 20, 2021. https://github.com/CJCascalheira/structural_stigma

[8] P. Cohen, J. Cohen, L. S. Aiken, and S. G. West, “The problem of units and the circumstance for POMP,” *Multivariate Behavioral Research*, vol. 34, no. 3, pp. 315–346, 1999, doi: 10.1207/S15327906MBR3403_2.

[9] J. Moeller, “A word on standardization in longitudinal studies: Don’t,” *Frontiers in Psychology*, vol. 6, p. 1389, 2015, doi: 10.3389/fpsyg.2015.01389.

[10] T. D. Little, *Longitudinal structural equation modeling (methodology in the social sciences)*. The Guilford Press, 2013.

[11] C. Ji and B. N. Cochran, “2020 LGBTQ specific substance use service survey: A study on the availability and perceived helpfulness of treatment programs,” Master’s thesis, University of Montana, 2021. [Online]. Available: https://scholarworks.umt.edu/etd/11727

[12] V. Hopkins, “It’s coming from inside the House (of Commons): Agenda control, accountability, and interest group lobbying in majoritarian parliaments,” *Governance*, vol. 33, no. 3, pp. 693–710, 2020, doi: 10.1111/gove.12454.

[13] M. Pickup and V. Hopkins, “Transformed-likelihood estimators for dynamic panel models with a very small T,” *Political Science Research and Methods*, pp. 1–20, 2020, doi: 10.1017/psrm.2020.30.

[14] M. Pickup, P. Gustafson, D. Cubranic, and G. Evans, “OrthoPanels: An R package for estimating a dynamic panel model with fixed effects using the orthogonal reparameterization approach,” *The R Journal*, vol. 9, no. 1, pp. 60–76, 2017, doi: 10.32614/RJ-2017-003.

[15] M. Arellano and S. Bond, “Some tests of specification for panel data: Monte Carlo evidence and an application to employment equations,” *Review of Economic Studies*, vol. 58, pp. 277–297, 1991.

[16] C. Hsiao, *Analysis of panel data*, 3rd ed. Cambridge University Press, 2014. [Online]. Available: https://doi.org/10.1017/CBO9780511754203

[17] J. Wooldridge, *Econometric analysis of cross section and panel data*, 2nd ed. South-Western, 2010.

[18] B. N. Cochran, K. M. Peavy, and J. S. Robohm, “Do specialized services exist for LGBT individuals seeking treatment for substance misuse? A study of available treatment programs,” *Substance Use & Misuse*, vol. 42, no. 1, pp. 161–176, 2007, doi: 10.1080/10826080601094207.

[19] B. H. Baltagi, *Econometric analysis of panel data*, 6th ed. Springer, 2021. [Online]. Available: https://doi.org/10.1007/978-3-030-53953-5

[20] J. Honaker, G. King, and M. Blackwell, “Amelia II: A program for missing data,” *Journal of Statistical Software*, vol. 45, no. 7, pp. 1–47, 2011, doi: 10.18637/jss.v045.i07.

[21] L. Keele and N. J. Kelly, “Dynamic models for dynamic theories: The ins and outs of lagged dependent variables,” *Political Analysis*, vol. 14, no. 2, pp. 186–205, 2006, doi: 10.1093/pan/mpj006.

[22] M. Pickup, *Introduction to time series analysis*, vol. 174. Sage Publications, 2014. [Online]. Available: https://dx.doi.org/10.4135/9781483390857

[23] X. Lu and H. White, “Robustness checks and robustness tests in applied economics,” *Journal of Econometrics*, vol. 178, pp. 194–206, 2014, doi: 10.1016/j.jeconom.2013.08.016.
